# Latitudinal variation and plasticity in response to temperature in *Geukensia demissa*

**DOI:** 10.1002/ece3.9856

**Published:** 2023-02-24

**Authors:** Theresa R. Erlenbach, John P. Wares

**Affiliations:** ^1^ Department of Genetics University of Georgia Athens Georgia USA; ^2^ Odum School of Ecology University of Georgia Athens Georgia USA

**Keywords:** Bivalvia, climate change, keystone species, management, Mytilidae, population diversity

## Abstract

As global temperatures warm, species must adapt to a changing climate or transition to a different location suitable for their survival. Understanding the extent to which species are able to do so, particularly keystone species, is imperative to ensuring the survival of key ecosystems. The ribbed mussel *Geukensia demissa* is an integral part of salt marshes along the Atlantic coast of North America. Spatial patterns of genomic and phenotypic divergence have been previously documented, although their link with coastal environmental variation is unknown. Here, we study how populations of *G*. *demissa* in the northern (Massachusetts) and southern (Georgia) portions of the species range respond to changes in temperature. We combine assays of variation in oxygen consumption and RNA transcriptomic data with genomic divergence analyses to identify how separate populations of *G*. *demissa* may vary in distinct thermal environments. Our results show differences in constitutive oxygen consumption between mussels from Georgia and Massachusetts, as well as shared and disparate patterns of gene expression across temperature profiles. We also find that metabolic genes seem to be a strong component of divergence between these two populations. Our analysis highlights the importance of studying integrative patterns of genomic and phenotypic variation in species that are key for particular ecosystems, and how they might respond to further changes in climate.

## Graphical abstract

With climate change impacting sea surface temperatures, it is imperative to understand how ecosystems, and the species in them, might respond. Here, we evaluate how temperature impacts two different populations of the ribbed mussel, *Geukensia demissa*, examining phenotypic differences in oxygen consumption, along with transcriptomic differences in expression and divergence. Our data provide interesting insights into how populations of a species might respond to a changing climate.

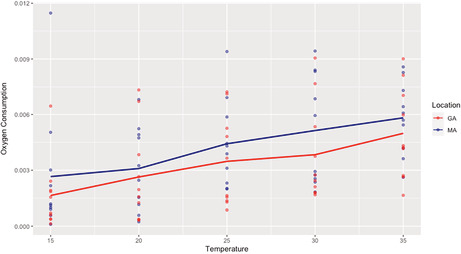

## 1 INTRODUCTION

Along broad latitudinal gradients, it is now typical to find that species are comprised of populations that are notably matched – via adaptation and acclimation – to their local environment (Broitman et al., 2021; Hice et al., 2012). In some cases, the populations sampled from a species' range may be quite obviously discrete (Kelly et al., 2012; Wares et al., 2021), or have the capacity for extensive gene flow and introgression (Coyle et al., 2019). Temperature tolerance is often a defining component for separating groups of organisms that will respond distinctly as the climate continues to warm. This is true when comparing different species (Popovic & Riginos, 2020) as well as geographically distinct populations within a species (Des Roches et al., 2018; Kelly et al., 2012).

A complementary response to adaptation or acclimation to a changing environment is that populations may shift in location as the climate warms (Palumbi et al., 2019; Sunday et al., 2012). In many cases, this may be a more likely response to climate change because individuals or their progeny can readily survive in suitable areas that may differ from what was their primary distribution only a few generations prior. This would be more effective in organisms with a mobile life stage, as they are able to adjust their distribution if their current habitat becomes unsuitable. Understanding how these responses – range displacement, adaptation, or acclimation – interact is key to recognizing what climate change will do to communities in all habitats (Peterson et al., 2019).

In many ways, coastal habitats are an important and simplified version of this problem. They are distributed largely in one coastal dimension, so that climate refugia are not defined by elevation but more directly by sea surface temperatures, salinity, and the subaerial temperature of low‐tide exposure (Wares & Skoczen, 2019). All species are subject to these dynamics, but it is of immediate importance to understand this set of dynamics in highly productive and protective autogenic habitats like coral reefs, seagrass beds, and coastal salt marshes (Power et al., 1996).

Along the Atlantic coast of North America, salt marshes are a key habitat that varies latitudinally in stresses ranging from ice scour and storm damage to heat and consumer‐driven risk (Bertness, 1999). The community diversity of these ecosystems is supported largely by one dominant foundation species, the cordgrass *Spartina alterniflora*. The cordgrass itself is supplied and stabilized by the ribbed mussel *Geukensia demissa*, with mussel aggregations ranging deep into the sediments and sometimes with dozens of individuals per mussel mound (Angelini et al., 2016). These bivalves stabilize the sediments and provide nitrogen‐ and phosphorus‐rich waste products from suspension feeding, which aids the growth and maintenance of salt marshes (Angelini et al., 2016). Given the importance of tidal salt marshes serving as nursery habitat for many commercial and recreational fish, and for carbon sequestration (Poppe & Rybczyk, 2021), finding elements that strengthen the stability and resiliency of salt marshes is critical.

Although *G*. *demissa* are free‐spawning in reproduction and their larvae spend weeks feeding in the water before recruitment as juveniles (Virgin et al., 2019), it has been recognized that latitudinal samples of *G*. *demissa* are not uniform. In fact, there are documented phenological shifts related to reproduction between mussels in New England and those in the southeast, as well as between mussels from distinct tidal heights in a marsh (Borrero, 1987). Díaz‐Ferguson et al. (2009) showed a statistical genomic difference, using mitochondrial sequence data, between populations of *G*. *demissa* in New England (Massachusetts) and those populations to the south (Virginia, Georgia, and Florida). This spatial pattern of genomic distinction is mirrored by many species across the Atlantic coast south of Long Island Sound (Altman et al., 2013; Bell et al., 2014; Wares, 2002; Zakas & Wares, 2012).

In addition to genomic differentiation between northern and southern populations of *G*. *demissa* (Díaz‐Ferguson et al., 2009), previous studies have indicated that there are also distinctions in protein expression between northern and southern populations of *G*. *demissa* (Fields et al., 2012), as well as seasonal variation (Fields & Eraso, 2020). Near the southern edge of their distributional range, temperatures in natural populations can exceed 45°C during emersion, which appears to be the maximum temperature the adults can survive (Jost & Helmuth, 2007). Variation in position on the marsh can also greatly influence a mussel's exposure risk, which itself varies latitudinally among populations (Julien et al., 2019).

Here, we study the potential population‐level response from this keystone species in a changing climate. First, we identify if there is variation in oxygen consumption across temperatures spanning typical summer sea surface temperatures between populations of *G*. *demissa* representing the northern portion (from Massachusetts) and southern edge (from Georgia coast) of this species' range. Then, we evaluate constitutive and temperature‐responsive variation in gene regulation between *G*. *demissa* from these two populations to identify patterns of transcriptomic divergence. These transcript sequences also provide insights into genomic differences between these two populations of ribbed mussels.

## 2 METHODS

Two distinct collections of *G*. *demissa* were used for this study. In May 2020, mussels were opportunistically collected from sites near the Marine Biological Laboratory in Wood's Hole, Massachusetts (41.576° N, 70.639° W) and near Savannah, Georgia (32.038° N, 81.048° W). These locations were chosen to span the recognized genomic and environmental distinctions between sites as well as pandemic availability for specimen collections. They vary considerably by mean monthly sea surface temperatures (average difference 10.8° ± 1.8°C, calculated from NOAA ERDDAP data from 2001–2019, see Figure S1), with maximum average temperatures in August (19.4°C in Massachusetts, 28.8°C in Georgia; see Villeneuve et al., 2021). Mussels were collected from marshes at elevations close to mean low tidal height. Specimens from 2020 contributed to the initial temperature‐focused transcriptomic analysis. The same locations/populations were sampled in 2021 to evaluate the physiological phenotype of these populations. The average living mass of mussels from both populations was 40.8 ± 14.3 grams.

### 2.1 Population‐based oxygen consumption

To evaluate the temperature‐based metabolism of mussels collected from Georgia and Massachusetts, we performed an experiment to quantify dissolved oxygen (DO) consumption. *G*. *demissa* specimens collected in June 2021 were acclimated to laboratory microcosm conditions for a minimum of 2 weeks before initiating the experiment (Thompson et al., 2012). Both populations of mussels (*n* = 10 from each) were maintained in Athens, GA in numbered mesh ‘pecan bags’ in controlled 30 L aquaria at 25°C for 2 weeks minimum upon receipt. Water was Instant Ocean maintained at 28‐30 ppt and pH 8–8.1; we maintained the water temperature in each tank with separate Teco T1000 heater‐chiller units. We fed the mussels ad libitum phytoplankton (Reef Phytoplankton, Seachem Laboratories) throughout the acclimation period, with 7.5 L water exchanges every 2 days. For the experiment, we used two OnSet temperature x DO loggers (Hobo U26‐001) for assessing metabolic rate associations with temperature.

Feeding stopped 2 days prior to the experiment. At the beginning of the experiment, the tank(s) were lowered to 15°C with adjustments to the next setpoint slowly overnight after each trial. For 10 individuals from each population, at each temperature point (15°, 20°, 25°, 30°, 35°), these individuals were evaluated for oxygen consumption for ~90 min (at 10 min intervals, discarding data from the first 20 min) in a small‐volume (800 ml) chamber containing a Hobo U26‐001 logger, an individual mussel, and a small stir bar. The temperature‐maintained system was positioned above a stir plate to maintain adequate water mixing without temperature adjustments (see Wares & Duffin, 2019). The first 20 min of chamber time was trimmed to account for acclimation to the tank prior to the assay.

To confirm the initial results, and ensure that they were not driven by the oxygen probe used for either chamber, oxygen consumption was repeated with collections of individuals (5 mussels from one population at a time) in a 10 L chamber (Tupperware). In these trials, both Hobo U26‐001 oxygen probes were included side by side, and the time of data collection was the same as before. These trials were only run at 20, 25, and 30°; at 25°, only one bulk sample of 5 mussels was used rather than both. This enabled simultaneous use of both DO loggers in the same 10 L container to ensure that no variation in result based on which logger was used.

Data on oxygen consumption were converted to VO2 as in Fly et al. (2012). The mass of each mussel was measured in three ways: the *gross* weight was taken with the whole living animal, ensuring that we could analyze any data if an individual died before the end of the experiment (none did). After the experiment, mussels were euthanized by freezing and then thawed to remove the body from the shell. The *shell* weight was recorded, along with the *pickled* body mass following 72 h of preservation in 95% ethanol, with a replacement of ethanol after 24 h. We evaluated oxygen consumption (VO2) using an additive general linear model with predictors of individual, temperature, and location. This analysis used the glmulti package (v1.0.8, Calcagno, 2020) in R 4.1.2 (R Core Team, 2022), assuming a Gaussian distribution.

Individuals used in this experiment were Sanger sequenced at the mitochondrial COI locus (using protocol and primers from Francis & Wares, 2022) to confirm identity and re‐assess mitochondrial divergence between MA and GA (as in Díaz‐Ferguson et al., 2009). For these data, we assessed sequence diversity and divergence using DNAsp v6.12.03 (Rozas et al., 2017).

### 2.2 Differential expression

We sought to identify if there are differences in gene expression when mussels from GA and MA are exposed to different temperatures. To do so, individual *G*. *demissa* were sampled in May 2020 from the spatial populations noted above. Under COVID‐19 campus research authorization, both populations of mussels (*n* = 36 each) were maintained in Athens, GA in numbered mesh ‘pecan bags’ in controlled 30 L aquaria at 25°C for 2 weeks minimum upon receipt (Thompson et al., 2012). Water was Instant Ocean maintained at 28‐30 ppt and pH 8–8.1; we maintained the water temperature in each tank with separate Teco T1000 heater‐chiller units. We fed the mussels ad libitum phytoplankton (Reef Phytoplankton, Seachem Laboratories) throughout the acclimation period, with 7.5 L water exchanges every 2 days.

After both populations had acclimated for a minimum of 2 weeks to laboratory conditions at 25°C, 5 mussels from each population were randomly selected, removed from the aquaria, and sampled for tissues; mantle tissue (see Gallardi et al., 2021; Malachowicz & Wenne, 2019; Yévenes et al., 2021) was sampled and stored in RNALater at −20°C. The remainder of tissues and shells were preserved in 95% ethanol and curated at the Georgia Museum of Natural History (Table S1). At this time, the two aquaria were either set to the experimental temperatures of 20°C (approximate mean August Sea Surface Temperature (SST) on coast of Massachusetts) or 30°C (approximate mean August SST on coast of Georgia; see Villeneuve et al., 2021), as elevated temperature has been determined as a contributing factor in change in abundance in confamilial mussels (Petraitis & Dudgeon 2020). Each aquarium contained five mussels from each population, for a total of ten mussels tested at each experimental temperature (20 and 30°C). Mussels were maintained at these temperatures for 2 weeks (Thompson et al., 2012). After 2 weeks, the remaining individuals from each population (Table S1) were again harvested for tissues for RNA isolation and preservation in ethanol.

RNA was isolated from individual mussels using Qiagen RNEasy preps as in Chandler and Wares (2017). We submitted RNA from each sample to Psomagen for quality assessment and sequencing. Libraries were prepared for each sample using the TruSeq stranded mRNA LT kit and sequenced on a single lane on the Illumina 2500 for paired‐end 150 bp reads. Three samples from Massachusetts had low RNA integrity numbers (one each from the three temperatures examined)  as well as one sample from Georgia representing 30°C. These were excluded from further analyses.

Reads were trimmed with fastp (Chen et al., 2018) and a transcriptome was generated for each sample with Oyster River Protocol (MacManes, 2018). Transcriptome completeness was assessed with the metazoan dataset in BUSCO v5 (Simao et al., 2015). A Massachusetts mussel (MA13‐30) had an extremely low BUSCO score (73.8%), so we excluded it from remaining analyses. We identified the GA control and MA control transcriptomes that had the best BUSCO score and used these as the references for the differential expression (DE) analysis. We chose to use a control from each population to ensure that we did not bias for a particular location and ensure confidence in the differential expression results due to pooled assemblies producing large numbers of duplicate sequences (see Discussion). We used cd‐hit‐est (Fu et al., 2012) to cluster each of the reference transcriptomes using the default identity threshold. Libraries for each sample were aligned to each reference transcriptome with Bowtie2 (Langmead & Salzberg, 2012). Salmon (Patro et al., 2017) was used to quantify the expression of RNA transcripts to each reference transcriptome using the fastq files of trimmed reads. We performed a differential expression analysis with edgeR (Robinson et al., 2010) using the quantified transcripts. We filtered read expression by only including those that had counts‐per‐million (CPM) greater than one in at least two of the libraries. This was performed twice, once with the best control GA transcriptome (GAC3) as the reference, and a second time with the best MA (MAC5) control transcriptome as the reference.

To identify constitutive differences in gene expression between the two populations (GA and MA), we compared the populations to each other, looking at differences between the controls (GAC vs. MAC) and between the two temperatures examined (GA20 vs. MA20 and GA30 vs. MA30). Responses to temperature were also evaluated through within‐population comparisons (GA20 vs. GA30 and MA20 vs. MA30). Differences in expression between comparisons were identified using glmTreat in edgeR (Robinson et al., 2010). Significant differences in expression were defined as having a FDR < 0.05. We identified homology to genes of significantly differentially expressed transcripts using the nt nucleotide data base in a blastx search. A transcript was only described if it was homologous to a *Mytilus* gene or other closely related bivalve. A time‐calibrated phylogeny of *Mytilidae* indicates that the split between the *Mytilus* genus and *Geukensia* was during the Devonian period, approximately 379.9 million years ago (Audino et al., 2020). Despite this divergence, the *Mytilus* genus is the most closely related species that is well annotated enough to provide insights into transcripts identified in our study. If this criterion was not met, the transcript was described as not having significant similarity to known proteins. We also performed a blast search of the two reference control transcriptomes to the other to identify homologous transcripts. This allowed us to identify transcripts between both DE analyses that were shared.

### 2.3 Population genetics

To identify divergence between and within these populations, we first generated coding sequence files with TransDecoder (Haas et al., 2013) using the individual transcriptomes. Single‐copy orthologs present in the coding sequence files were identified by OrthoFinder (Emms & Kelly, 2019) and aligned with MAFFT v7.470 (Katoh & Standley, 2013). Orthologs are defined in OrthoFinder by first performing a BLAST all‐versus‐all search, comparing each sample against each other. The best hits between all individuals are then normalized based on gene length and phylogenetic distance. Thresholds for similarity are defined by the reciprocal best length‐normalized hit, and this threshold must be hit for a sequence to be included as an orthogroup (Emms & Kelly, 2015). We calculated divergence statistics (*S*_nn_, Tajima's D, π per site, and *d*_A_) on these orthologs using the R package PopGenome (Pfeifer et al., 2014). A total of 1000 permutations were performed in R to determine significance. These permutations were done by randomly sampling each population (fourteen individuals for Georgia and eleven for Massachusetts) and recalculating the statistic 1000 times. Significance was determined if the observed statistic was greater than 95% of the null distribution generated from the permutations.

As metabolic loci can be targets of selection (Marden, 2013), we used nineteen metabolic genes previously identified (Skibinski & Ward, 2004) and pulled their nucleotide sequences in *Mytilus galloprovincialis* from NCBI. A fasta file of all orthologs from OrthoFinder was generated by concatenating the longest sequence from each ortholog's fasta file. We then created a BLAST database for this ortholog fasta file. The FASTA files for each metabolic gene identified in *M*. *galloprovincialis* were used as the query for a tblastx search against the ortholog fasta file to identify which ortholog correlated with each metabolic locus of interest. In addition, the M7 Lysin sequence, an important gametic recognition protein identified in sperm in *Mytilus*, was downloaded from UniProt (Accession ID:I7IG91). We included this additional locus since it has been found to be divergent between allopatric populations of *Mytilus* (Riginos & McDonald, 2003) and we were interested to identify if similar patterns of divergence are occurring in *G*. *demissa*. This gene was used as a query for a tblastn search of the ortholog fasta files. Each tblastn search required an e‐value of at least 10 e‐05 for a match. Orthologs with the best hit for each locus were aligned with MAFFT v7.470 (Katoh & Standley, 2013). *d*_A_, Tajima's D, π per site, F_st_, and *S*_nn_ were calculated for metabolic loci and M7 Lysin transcripts in PopGenome (Pfeifer et al., 2014). A total of 1000 permutations were performed on these statistics in R to determine significance as described above.

To ensure that loci of interest were indeed divergent and not an artifact of mis‐alignment or noise, we examined alignments for transcripts that fell within both the top 5% of *d*_A_ and *S*_nn_ more closely, where we recognized that some alignments were not aligned as cleanly as others (e.g., gaps introduced by lower quality regions, length variation). The alignments for this smaller dataset were downloaded, manually curated, and re‐analyzed in Geneious v11.1.5, recognizing that alignment quality could bias the analytical results. Alignment gaps were evaluated by eye, particularly near homopolymers, and individuals missing >20% of the inferred transcript length were excluded from further analysis. The Geneious de novo assembler was used to dissolve and reassemble the reads from each transcript, allowing gaps with a maximum per read of 5%, maximum gap size of 5, and mismatches per read of 20% with no merging variants. Only alignments with at least 15 sequences and a minimum of five individuals for each population were analyzed. Divergence metrics (*S*_nn_, *d*_A_, and *d*_N_/*d*_S_) were calculated in DNAsp v6.12.03 (Rozas et al., 2017). Homology to known proteins for the non‐metabolic loci transcripts analyzed here was done with blastx and the nr database. We only described transcripts where the top hit was a *Mytilus* gene.

## 3 RESULTS

### 3.1 Respirometry

**FIGURE 1:**
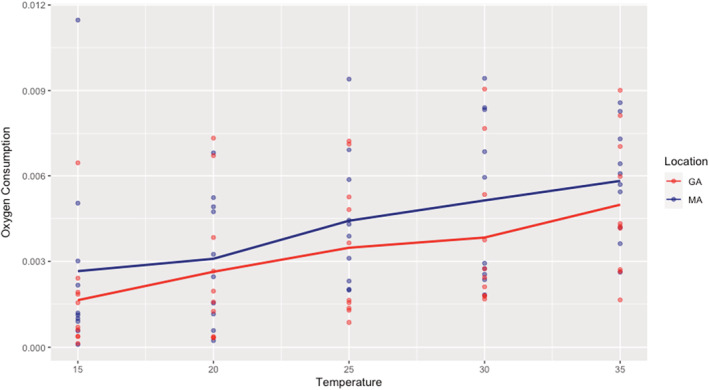
Temperature performance distributions for *G. demissa* from GA and MA based on oxygen consumption (μmol O_2_ g^−1^ h^−1^) at 5 temperatures (indicated in Celsius). Lines connect mean values across individuals for each population.

We evaluated if there are differences in dissolved oxygen consumption between mussels from Georgia and Massuchetts when exposed to increasing temperature. Figure 1 shows a consistent ~31% increase (average across temperatures, using the core observations and the pickled mass) in oxygen consumption across temperatures for the mussels from Massachusetts relative to Georgia. Our GLM using individuals, location, and temperature as predictor variables finds that temperature strongly drives oxygen consumption (*p* < .0001; *t* = 4.726), and location also appears important (*p* = .026; *t* = 2.253). This effect is only stronger if the gross weight of the mussel is used, although there is no site distinction in body mass per shell mass (JPW results not shown).

We repeated the dissolved oxygen consumption experiment to ensure that results were not due to the oxygen probe used. The two oxygen probes generated ΔO_2_ values between 10 min time points that were always ≤0.01 mg/L of each other. As in Figure 1, the difference between populations in oxygen consumption at 20° was negligible (MA values ranging from 97.8 to 113.1% of GA values), but was at least (depending on mass index used) 27% higher in MA than GA at 25°, and at least 55% higher in MA than GA at 30° (Table S2).

We assessed genomic divergence at the mitochondrial COI locus for the specimens in the dissolved oxygen experiment (NCBI OL893108‐OL893127). The diversity and divergence were comparable to that found in Díaz‐Ferguson et al. (2009); in that study, the GA specimens were collected at Sapelo Island, GA (31.389012, −81.279469) and the MA specimens from near Gloucester, MA (42.609463, −70.673723). Combining those data with the data derived from this experiment, S_NN_ is 0.8928 (*p* = .000) and F_ST_ is 0.263. Looking only at the data from our DO experiment – bearing in mind that these are distinct mussels from those in the RNA sequence data – S_NN_ is 0.7563 (*p* = .002) and F_ST_ is 0.229.

### 3.2 Differential gene expression

Individuals collected for data in this paper are listed in Table S1 with their GMNH accession numbers, source location, treatment (25°/20°/30°), NCBI SRA accessions, library RIN values, and read depth. For all libraries, Q20(%) was 96.5 or higher. All data are available in the NCBI BioProject PRJNA729970. There were 12 individuals sequenced for Massachusetts across the three temperatures and 14 for Georgia.

To identify differences in expression between the two populations, we performed two differential expression analyses, using a control transcriptome from each population as the reference. This was done to avoid pooling reads and creating many false transcripts from a bulk transcriptome and ensure that we did not bias toward one population. The GA control reference transcriptome had 141,642 contigs with a total length of 93,980,237 bases (N50 971) and a BUSCO score of 94.8%, with 88.4% of these single‐copy. The MA control reference transcriptome had 138,198 contigs with a total length of 89,251,500 bases (N50 925) and a BUSCO score of 93.9%, with 86.7% of these single‐copy. Individual libraries for each sample were aligned to each reference transcriptome and their expression was quantified for 54,681 and 54,898 inferred transcripts for GA and MA, respectively. Mapping efficiency of each library as reported by bowtie2 is found in Table S1. Significant differences in expression were identified by transcripts having a false discovery rate (FDR) < 0.05. We examined the relationship of the samples to each other using a PCA plot, where PC1 (10%) most likely represents the experimental temperatures examined in this study and PC2 (8%) the two populations (Figure S2).

To identify constitutive differences between the two populations, we examined the expression differences between the controls and the two experimental temperatures from the two populations (contrasts GAC / MAC, GA20 / MA20, and GA30 / MA30). We find that overall, there are few constitutive differences between the two populations (Table 1; Figure S3). Differentially expressed transcripts shared between both reference assemblies had little homology to known proteins, most likely due to shorter sequences. One transcript, however, had greater expression in Massachusetts mussels using both reference transcriptomes and was homologous to DDX5, which is an ATP‐dependent RNA helicase. Some transcripts were differentially expressed, but did not share homology with the other transcriptome. Transcripts unique to the MAC reference had homology to a taurine transporter (GA20vsMA20 logFC = 2.25 [FDR < 0.005]) and a tubulin beta chain (GA20vsMA20 logFC = −2.45 [FDR < 0.005]). No other transcripts had homology to any known protein based on our criteria (see Methods) or any significant BLAST hits.

**TABLE 1:** Numbers of transcripts with constitutive differences in expression between Georgia and Massachusetts using either the GA or MA control reference transcriptome.

Comparison	GAC reference transcriptome		MAC reference transcriptome	
	Higher expression in Georgia	Higher expression in Massachusetts	Higher expression in Georgia	Higher expression in Massachusetts
GAC vs. MAC	4	3	4	11
GA vs MA (20°C)	4	1	3	5
GA vs MA (30°C)	4	0	1	0

**FIGURE 2:**
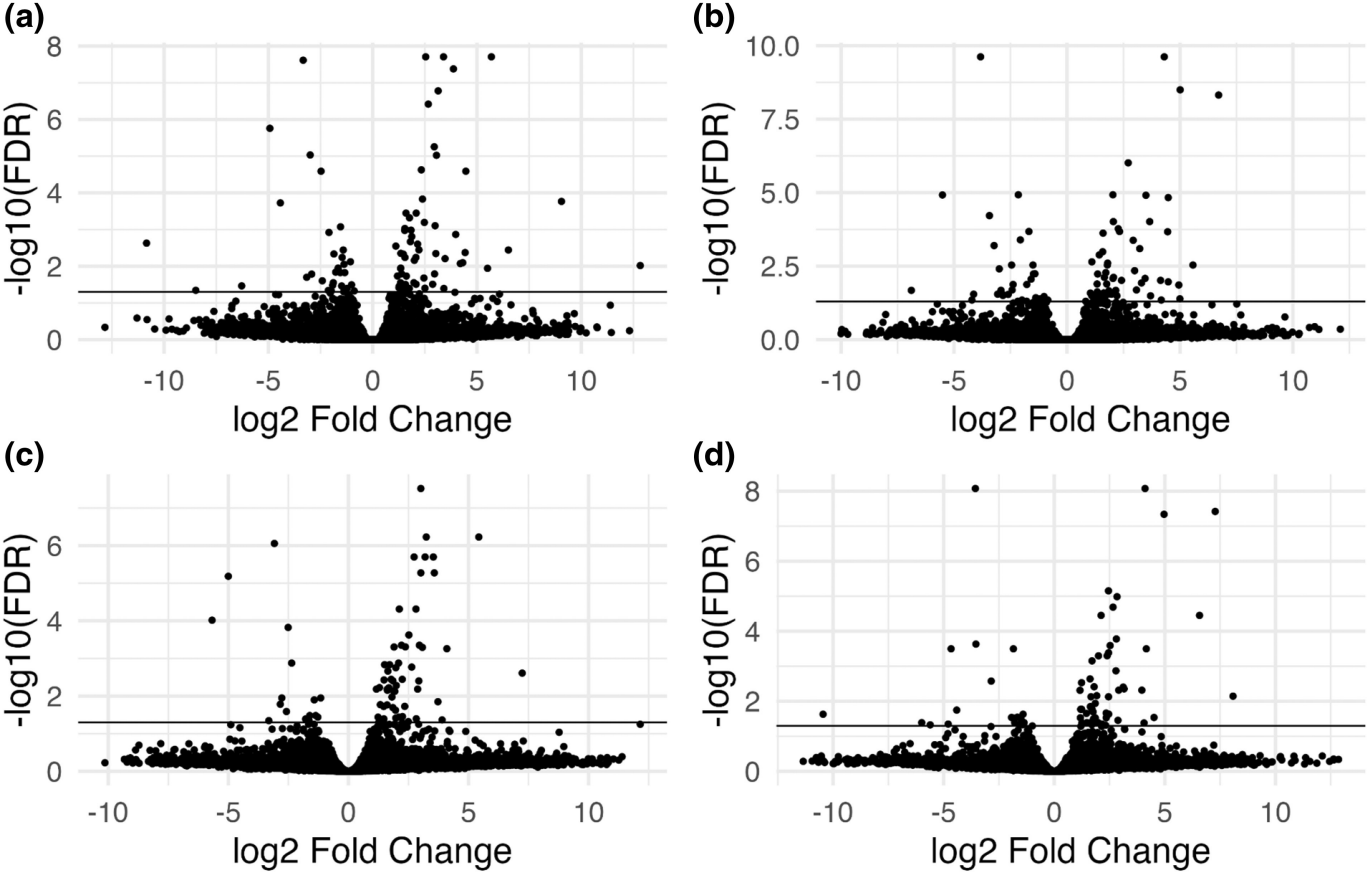
Within‐population differential transcript expression between exposure to 20° and 30°. Log2 Fold Change higher than 0 indicates higher expression at 20° and log2 Fold Change below 0 is higher expression at 30°. (a). Results for the Georgia population (GA20 / GA30) with GAC3 reference transcriptome. (b). Results for the Georgia population (GA20 / GA30) with MAC5 reference transcriptome. (c). Results for the Massachusetts population (MA20 / MA30) with GAC3 reference transcriptome. (d). Results for the Massachusetts population (MA20 / MA30) with MAC5 reference transcriptome. Significance is indicated at FDR < 0.05.

We then examined how temperature affects the expression within each population. In general, there were more transcripts expressed at 20 than 30°C for both populations and methods (Table 2; Figure 2). Within Massachusetts, five transcripts were homologous to tubulin alpha and beta chain proteins, all of which had higher expression of these transcripts at 20°C [FDR < 0.005]. Several transcripts also had homology to splicing factors and had higher expression at 20°C, including heterogeneous nuclear ribonucleoprotein (logFC = 3.01[FDR < 0.005] and logFC = 2.83[FDR < 0.005] for GA and MA reference, respectively), serine‐/arginine‐rich splicing factor 2 (logFC = 5.42[FDR < 0.005] and logFC 7.27[FDR < 0.005]), and a spliceosome RNA helicase (logFC = 1.77[FDR < 0.005] and logFC = 1.8[FDR < 0.005]). One transcript was homologous to a glutathione S‐transferase (logFC = 2.51[FDR < 0.005] and logFC = 2.52[FDR < 0.005]). A transcript homologous to an xBox binding protein was also differentially expressed, but with higher expression at 30°C (logFC = −1.42[FDR < 0.05] and logFC = −1.41[FDR < 0.05]). Lastly, an elongation factor 1 alpha homolog had higher expression at 20°C (logFC = 1.95[FDR < 0.05] and logFC = 4.5[FDR < 0.05]). All other transcripts did not have homology to known proteins.

**TABLE 2:** Number of transcripts showing temperature‐based effect on expression within Georgia and Massachusetts based on either the GA or MA control reference transcriptome.

Comparison	GAC reference transcriptome		MAC reference transcriptome	
	Number of transcripts with higher expression at 20°C	Number of transcripts with higher expression at 30°C	Number of transcripts with higher expression at 20°C	Number of transcripts with higher expression at 30°C
GA 20°C vs GA 30°C	62	33	55	35
MA 20°C vs MA 30°C	56	18	54	19

Georgia mussels also had many differentially expressed transcripts homologous to tubulin proteins, all of which had higher expression at 20°C [FDR < 0.005]. Additionally, splicing factors were found to be differentially expressed, including a heterogeneous nuclear ribonucleoprotein (logFC = 2.54[FDR < 0.005] and logFC = −2.15[FDR < 0.005]), serine‐/arginine‐rich splicing factor 2 (logFC = 5.69[FDR < 0.005] and logFC = 6.7[FDR < 0.005]), and splicing factor A2AF (logFC = 2.99[FDR < 0.005] and logFC = 3.21[FDR < 0.005]). A glutathione S‐transferase homology had greater expression at 20°C (logFC = 1.8[FDR < 0.005] and logFC  = 1.79[FDR < 0.005]), along with a 5′‐3′ exoribonuclease (logFC = 1.34[FDR < 0.005] and logFC = 1.35[FDR < 0.005]), and a SEH1‐like nucleoporin (logFC = 1.33[FDR < 0.005] and logFC = 1.85[FDR < 0.005]). Higher expression at 30°C was found for an x‐Box‐binding protein (logFC  = −1.41[FDR < 0.005] and logFC = −1.41[FDR < 0.005]), a DDX5 homolog (logFC = −1.37[FDR < 0.005] and logFC = −1.5[FDR < 0.005]), and an anaphase‐promoting complex subunit 11 homolog (logFC = −1.36[FDR < 0.005] and logFC = −1.33[FDR < 0.005]). Homology was not found for the remaining transcripts.

### 3.3 Population divergence

We calculated the population genetic statistics for these populations using 833 single‐copy orthologs that were identified in the individual transcriptomes. We note that these are synthetic transcripts as we do not have an annotated genome to provide more accurate full‐length sequences, or include within‐individual polymorphism, for these analyses. The number of sites analyzed in each ortholog varied, with the average being 1205 nucleotides (min of 321 and max of 6008). Our population sample sizes were 14 for Georgia and 11 for Massachusetts.

**FIGURE 3:**
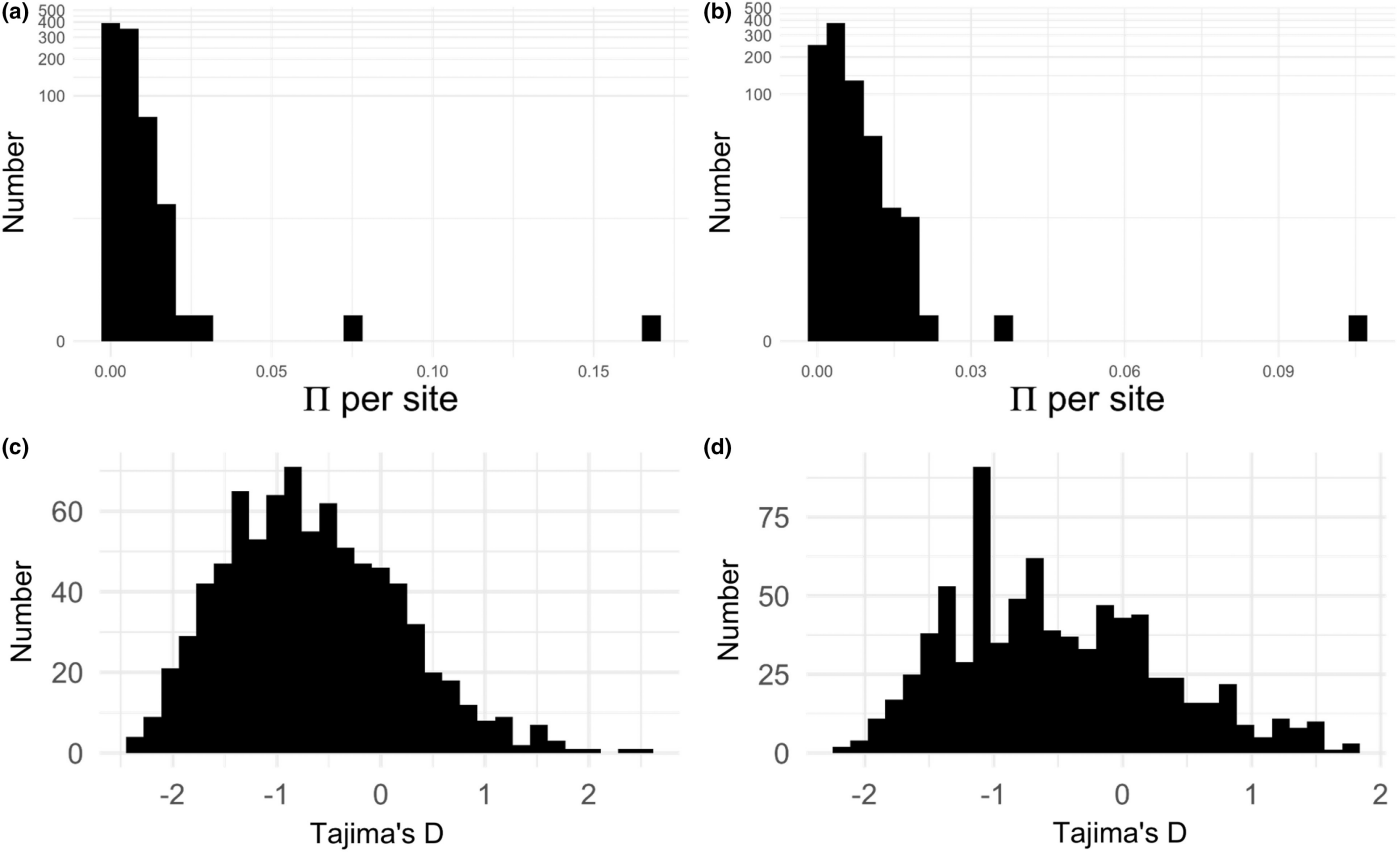
Within‐population genomic diversity statistics. Nucleotide diversity (π) among single‐copy orthologs from (a). All 14 individuals from Georgia and (b). All 10 individuals from Massachusetts. The y‐axis for panels a and b are log transformed. Distribution of Tajima's d in single‐copy orthologs from (c). Georgia and (d). Massachusetts.

We first calculated statistics to analyze the diversity patterns within the populations (π per site and Tajima's D). Nucleotide diversity within the populations was relatively low, with most transcripts falling near zero (Figure 3a,b). The mean π per site for Georgia orthologs was 0.0043, with a max of 0.167. For Massachusetts orthologs, the mean π per site was 0.0041, with a max of 0.105. The distributions of π per site between Georgia and Massachusetts are not statistically different from one another (Kolmogorov–Smirnov Test D = 0.057; *p* = .12). In the distribution of Tajima's D (Figure 3c,d), we see a strong negative shift, indicating an excess of low‐frequency polymorphisms. There are some orthologs, however, that do have a high Tajima's D, which indicates that there are some variants that are in high frequency in the population and a concordant lack of rare alleles. Our distributions of Tajima's D are statistically different from one another (Kolmogorov–Smirnov Test D = 0.095; *p* = .0011).

**FIGURE 4:**
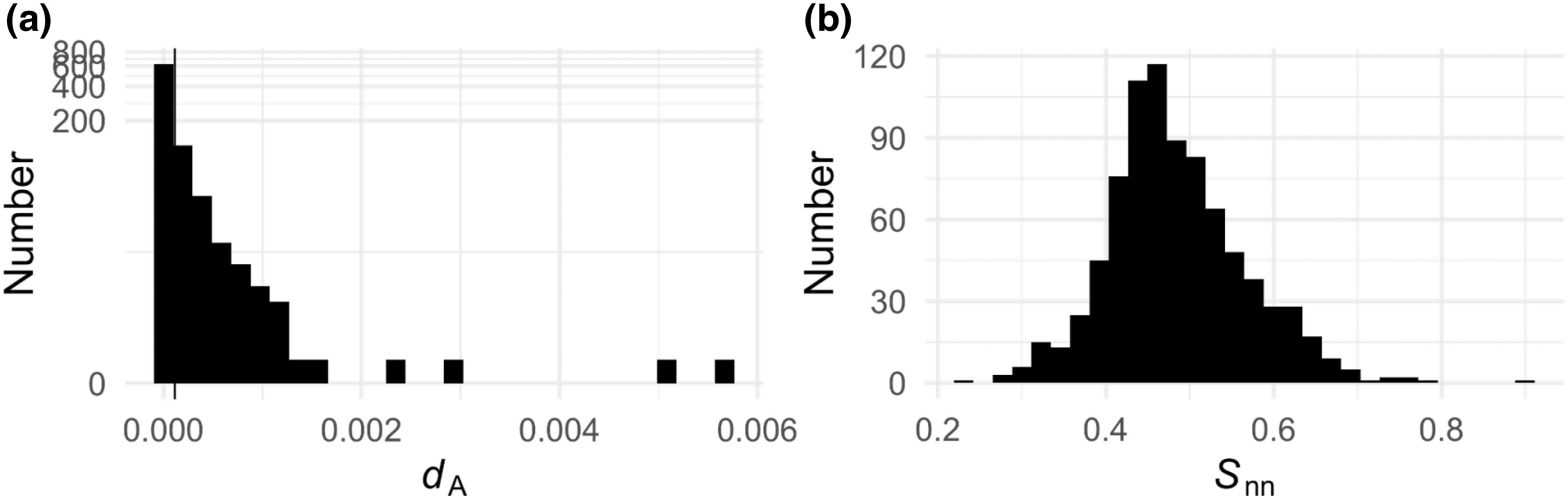
Between‐population statistics of divergence. (a). Nei's net nucleotide divergence, d_A_, per single‐copy ortholog. The y‐axis is log‐transformed. (b). Distribution of S_nn_ per single‐copy ortholog; the null value for 2 populations is expected to be 0.5, variation around that is largely sampling error, but extreme positive values are considered further in the text.

Between‐population divergence for all single‐copy orthologs was examined by calculating *d*_A_ and *S*_nn_. Net nucleotide divergence across the populations had a mean *d*_A_ of 0.00011 with a max *d*_A_ of 0.0056 (Figure 4a), indicating low average genetic divergence between populations. The *S*_nn_ statistic, which describes how often the most similar sequences are from the same geographic space, has a mean of 0.48 (Figure 4b). When this statistic is near 1, the two populations sampled are expected to be highly differentiated; however, at an *S*_nn_ of one‐half, the populations are effectively panmictic (Hudson, 2000). With a mean of 0.48, these single‐copy orthologs indicate that the populations sampled are not highly differentiated, on average. There are a few transcripts, however, that have a higher *S*_nn_ statistic that show more differentiation than the rest of the orthologs, some of which are statistically significant and examined further.

To evaluate orthologs of interest, we set criteria to determine what orthologs could be most biologically relevant. We looked at the top 5% of orthologs for *d*_A_ and *S*_nn_ and identified orthologs shared between these two statistics. In addition, we identified 19 metabolic loci based on previous work (Skibinski & Ward, 2004) in our orthologs. Between the two sets (metabolic loci and shared between top 5% of *d*_A_ and *S*_nn_), there were 31 alignments we analyzed for divergence (Table S3). These alignments were visualized in Geneious and any problematic alignments were trimmed (see Methods) before recalculating divergence statistics in DNAsp (Table 3). We find that 12 of these 31 orthologs have a significant *S*_nn_, of which amylase has a value of 0.947 (*p* = .001). The distribution of the *S*_nn_ statistic for the manually curated loci follows that of the entire dataset, with most loci near 0.5 and the significant ones falling near the high‐end tail of the distribution (Tables 3 and S2). Interestingly, our best match to the *Mytilus* M7 lysin, a gametic recognition protein, also has a high *S*_nn_ (0.892; *p* = 0) and *d*_A_ (0.001), as did phosphoglucomutase (0.69 and 0.00093 for *d*_A_ and *S*_nn_, respectively). None of the other metabolic loci examined here were significant for *S*_nn_, and many had very little divergence between the two populations. These included albumin, purine nucleoside phosphorylase, isocitrate dehydrogenase, and others, indicating that these are most likely well conserved and not experiencing strong divergent selection. We did assess values of *K*a/*K*s for these loci and found that all were unremarkable, for example, the highly divergent amylase region *K*a/*K*s = 0.136 (Table 3). Interestingly, however, three loci exhibit statistically exceptional values of Tajima's D when samples are combined – including amylase (1.852) and glutamate dehydrogenase (2.628) with extremely positive values. Remembering that these are synthetic haplotypes being analyzed, which may not accurately account for heterozygosity within individuals, more work will be necessary to interpret these dynamics. We note that more highly divergent orthologs are also less likely to be recovered from other distantly related bivalve genomes.

**TABLE 3:** Divergence statistics for metabolic loci and orthologs of interest following additional alignment curation, listed in order of S_nn_ statistic.

Transcript	N [GA]	N [MA]	DNAsp bases	*S*_nn_	*S*_nn_ (p)	*d*_A_	*K*a/*K*s	Taj D
Amylase	9	10	973	0.947	0.001	0.01522	0.136	**1.852** ^(*)^
OG0017382	14	11	1263	0.892	0	0.00184	0.162	0.353
OG0016831	14	11	3045	0.85667	0	0.00094	0.139	0.112
OG0016789	14	11	897	0.85477	0	0.00089	0.07	−1.907*
Lysin*	12	7	927	0.78947	0.008	0.00347	0.189	0.386
OG0017590	14	11	408	0.73111	0.002	0.00057	0.328	−1.266
OG0016625	14	11	682	0.724	0.007	0.00099	0.151	−0.233
OG0016844	14	9	402	0.7029	0.024	0.00149	0.098	0.613
Pgm	14	11	790	0.69667	0.023	0.00093	0.081	0.292
OG0017668	14	11	1527	0.68533	0.014	0.00313	0.117	−0.309
OG0016708	14	11	774	0.67667	0.037	0.00122	0.127	−0.379
OG0017577	14	11	1422	0.67333	0.029	0.00103	0.009	−0.673
G6PDH	7	8	1657	0.62667	0.16	−0.0001	0.012	−0.612
AcidPhos	8	7	148	0.564	0.158	0.0045	0.524	0.513
OG0016809	14	11	726	0.5535	0.238	0.00044	0.031	0.709
Glutamate Dehydrogenase	14	11	245	0.5401	0.157	0.00113	0.082	2.628**
iDH	13	9	701	0.52543	0.278	−0.00006	0.000	−0.161
MPI	14	11	1065	0.51219	0.381	0.00021	0.260	0.145
LDH	11	5	1208	0.5	0.666	−0.0009	0.028	−0.287
HK	11	10	3667	0.4881	0.465	0.00015	0.058	−1.32
Albumin	11	11	1174	0.47608	0.55	0	0.003^	−1.729
Purine Nucleoside Phosphorylase	14	11	859	0.46462	0.626	0.00001	0.024	−1.017
Alkaline Phosphatase	12	9	1417	0.44048	0.638	−0.00017	0.100	−0.606
Isocitrate Dehydrogenase	14	11	1110	0.4	0.819	0.00016	0.015	−0.708
GPI	12	8	1224	0.275	0.961	−0.00052	0.087	−0.418

*Note*: Transcripts beginning with OG are those shared between the top 5% of Snn and dA. Transcripts named are metabolic loci. Significance denoted as (*) between 0.05 and 0.1, * less than 0.05, and ** less than 0.01. ^ indicates observation with Ks = 0.

Bold values are those with significant values of S_nn_.

## 4 DISCUSSION

Here, we surveyed physiological, transcriptomic, and genomic variation within the distributional range of *Geukensia demissa* – from close to the southern range boundary of the species (Georgia) to a point 1500 km to the north (Massachusetts) that is environmentally quite distinct (Figure S1). We find that there are multiple lines of evidence for evolutionary divergence that may be functionally important. Although our common garden experiments cannot rule out maternal or other acclimatory effects (see Sanford & Kelly, 2011), we show that there are both shared and divergent patterns of gene expression across a range of temperatures for individuals from these two populations, and a consistent difference in oxygen consumption across a range of temperatures that suggests a heritable difference as well as observed genomic divergence across latitudinal samples. The physiological observation is consistent with other studies suggesting frequent countergradient variation in traits associated with growth and maturation on the Atlantic coast (Levinton, 1983; Villeneuve et al., 2021; Yamahira & Conover, 2002). The transcriptomic and genomic contrasts, however, exhibit more subtle components of divergence between the two populations – across a large portion of the spatial range of *G*. *demissa* – and more work will be necessary to identify specific regional and mechanistic drivers for this variation (Hice et al., 2012).

Our RNA sequence data were used both to determine how generalizable the genomic divergence between these populations is and to see how transcription patterns separate these northern and southern populations of *G*. *demissa*. This combined use of data led to a number of interesting insights. Methodologically, we had attempted to first assemble a pooled reference transcriptome, using reads from all individuals. This led to overestimating the number of transcripts and very few that were differentially expressed, most likely due to alleles being aligned in different places due to multiple transcripts being very similar to each other. We attempted to remedy this with a clustering analysis, but there were still many transcripts that were very similar but not clustering together. These attempts helped us to discover that these two populations are divergent enough that building a pooled transcriptome of all reads was going to give false results and impact our interpretation of temperature on the mussels. To ensure that we still captured diversity between the two populations, we then decided to run our differential expression analysis using two different reference transcriptomes, which were the best control transcriptome from each population (GAC3 and MAC5). This reduced the number of transcripts in the assembly and brought more confidence in identifying homologs between the two populations. From there, we were able to identify differentially expressed loci that were affected by temperature on both populations. Our results were quantitatively similar to those obtained from our previous efforts (not shown).

Our differential expression analysis found more transcripts differentially expressed at the colder experimental temperature (20°C) within the populations (Table 2). Five transcripts had homology to tubulin proteins, which are an important component of the eukaryotic cytoskeleton. There was similarity to both ɑ‐ and β‐ tubulin genes, both of which are necessary to form long microtubule filaments, with *G. demissa* exposed to the lower temperatures having higher expression of these. In yeast, lower temperatures (10°C) can decrease rates of microtubule polymerization and depolymerization (Li & Moore, 2020). A differentially expressed transcript homologous to N‐acetyltransferase 9‐like protein (OG0017590) was in the top 5% of *d*_A_ and *S*_nn_ (0.73 and 0.00057 for *S*_nn_ and *d*_A_, respectively). These proteins are important for microtubule stability and cell survival in other species, like *Drosophila* (Mok & Choi, 2021). There could be an interaction between this differentiating transcript and the differentially expressed tubulin proteins where the decreased temperature is resulting in changes in tubulin polymerization.

We also found serine and arginine‐rich splicing factors to be differentially expressed. In plants, cellular homeostasis and thermotolerance can be impacted by differential splicing of genes under high temperatures. A study in *Solanum lycopersicum* showed that the expression of these splicing factors was impacted in response to heat stress (Rosenkranz et al., 2021). Oxidative stress has also impacted *Crassostrea virginica*, the Pacific oyster, where there is higher expression of an alternative splice of a highly conserved mitochondrial inner‐membrane oxidase (Liu & Guo, 2017). Oxygen consumption of *G*. *demissa* from Massachusetts was significantly higher than those from Georgia, and this could be a result of selective pressures to maintain oxygen consumption in cooler temperatures and increase efficiency during a shorter growing season (countergradient variation). Although we do not have environmental dissolved oxygen levels for either collection site, it would be interesting to identify if either of these populations is experiencing these stressors that could lead to a need for selection on genes that will respond to them.

To identify biologically relevant transcripts diverging between our two populations, we set conservative criteria for the population genetic statistics and the transcripts we described. Only 10 transcripts met these criteria, half of which did not have homology to *Mytilus* proteins. Those that did include the tubulin‐associated protein described above, as well as TRIM71 (OG0016789), UBA1 (OG0016831), and a minichromosome maintenance complex‐binding protein (OG0017668). TRIM71 and UBA1 are both associated with ubiquitin. In particular, UBA1 is involved in a variety of ubiquitin pathways, particularly in protein homeostasis (Groen & Gillingwater, 2015). Mutants of UBA1 have been shown to be temperature‐sensitive, with a particular mutation leading to the ubiquitin system failing in the nucleus (Sugaya et al., 2014). In plants, the ubiquitin process plays an important role in controlling and regulating any transcriptional changes that are needed to respond to abiotic stressors, such as increased temperature and anaerobic conditions (Lyzenga & Stone, 2012). Notably, in Pacific oysters, ubiquitination is most likely a conserved response to ocean acidification. When examining partial pressures of CO_2_, it was found that exposure to elevated levels caused proteins associated with the cytoskeleton, such as actin isoforms, and oxidative stress to be upregulated (Tomanek et al., 2011). Analysis of gonad methylomes in Pacific oysters also showed genes associated with protein ubiquitination to be differentially methylated in response to differing pH levels (Venkataraman et al., 2022). Here, we found that glutathione S‐transferase was upregulated in GA and MA at 20°C, and higher activity of this protein has been found in *M*. *edulis* extracted from sites with high pollution (Fitzpatrick et al., 1997), which can alter pH levels. The differential expression of these genes that have been shown to be involved in response to environmental stressors could be indicators of how *G*. *demissa* will respond to a changing climate. With pollution contributing to ocean acidification, and the importance of this bivalve in coastal environments, it is imperative to identify if differing pH and pollution levels are contributing to some of the divergence we see between these two populations and future studies to examine how these factors might impact this species response to rapidly increasing ocean temperatures and acidification.

Selection on genes involved in metabolism has been seen previously in other species that have populations in different climates (Deng et al., 2019; Marden, 2013; Paraskevopoulou et al., 2019; Skibinski & Ward, 2004). *G*. *demissa* could be experiencing selection pressure on metabolic functions, as there are some loci with sequence differences that could be attributed to geographic separation. Amylase, a metabolic gene important for digestion, had a significant *d*_A_ and *S*_nn_ (0.015 and 0.947 for *d*_A_ and *S*_nn_, respectively; *p* < .001); however, *d*_N_/*d*_S_ is not exceptional (0.136). This enzyme has been found to be sensitive to changes in feeding pattern in other bivalve species (Albentosa & Moyano, 2008) and polymorphisms of this enzyme are associated with differences in growth rate (Prudence et al., 2006). Amylase activity has also been found to be lower in mussels (*Mytilus californianus*) exposed to warmer temperatures in both high and low tide exposure (Pham et al., 2023).

In our data, sequences for phosphoglucomutase were also highly divergent (0.696 for *S*_nn_; *p* < .05). In *Mytilus*, phosphoglucomutase allele frequencies have been found to be different in individuals exposed to elevated water temperatures (LeBlanc et al., 2010), and it has previously been shown that this gene has alleles that are heat sensitive (Theisen, 1978). Alleles of these particular metabolic genes could be segregating between different populations of *G*. *demissa* in response to varying climate conditions. Similar studies on the Atlantic coast have also shown high levels of heterogeneity in terms of genomic differentiation, such as the silverside (*Menidia menidia*) with four large genomic regions that carry highly divergent diversity (Wilder et al., 2020). The type of allele that segregates in *Geukensia* at metabolically important loci could affect how these populations are able to survive in response to increased temperatures as climate change continues. Although we do not see strong signatures of selection, the divergence at some loci suggests that transient allopatry along with selection could have driven some of this evolutionary pattern.

We also find that there could be slight selection on proteins important for recognition between mussels. M7 lysin, for example, is an important c‐type lectin in the mussel *Mytilus* that has shown positive selection in allopatric populations of *Mytilus* taxa (Riginos & McDonald, 2003). This gametic recognition protein is important for sperm to dissolve the egg vitelline coat, which can then allow for fertilization (Vacquier, 1998). We identified a sequence very similar to *Mytilus* M7 lysin in our samples – including a match to the key QPD motif noted in Takagi et al. (1994) – and found this sequence to be significantly diverged between Georgia and Massachusetts (*S*_nn_ = 0.789; *p* = .008 and *d*_A_ = 0.00347; *p* < .05). If our two populations are diverging due to the large geographic distance between them, it would not be surprising to find that a gametic recognition protein is as well (Popovic et al., 2014). This could largely be due to selection enforcing recognition between the sperm and egg as seen in many other marine organisms, such as the sea urchin *Diadema* (Geyer et al., 2020). However, if there are traits specific to either of the two populations of *G*. *demissa* that provide an adaptive advantage as climate shifts, this could theoretically lead to the populations diverging from each other and the need for selection to reinforce recognition for mussels also from that population.

We know from earlier mitochondrial divergence studies that the Massachusetts populations are statistically divergent from other sampled *G*. *demissa* diversity as far north as Gloucester Point, Virginia (37.25° N; Díaz‐Ferguson et al., 2009), so the focus of further investigation will be in this northern Mid‐Atlantic Bight region of strong genomic divergence across many coastal taxa (Altman et al., 2013; Bell et al., 2014; Wares, 2002). Here, our focus was on the large temperature differential between MA and GA, which would be considerably lower for population contrasts north of Cape Hatteras, NC (Figure S1). However, the physiological divergence may or may not be coincident – perhaps it has to do with consistent shifts in phytoplankton availability in northern coastal waters. Or, we may have found an example of “latitudinal compensation,” where elevated physiological rates are found at higher latitude populations (Villeneuve et al., 2021). If so, countergradient variation is thought to allow for faster growth in a shorter “optimal” season (Levinton, 1983; Yamahira & Conover, 2002). It will be difficult to separate these two effects on response physiology in *G*. *demissa* in natural populations (Huey & Kingsolver, 2019), but that will be important to understand as climate warming continues (Villeneuve et al., 2021).

We note that static temperature experiments may tell us less than we would hope about organismal response to environmental variation (Marshall et al., 2021), and in fact constant submersion itself may limit the inference that can be made for studying physiological responses in intertidal organisms (Nancollas & McGaw, 2021). In particular, subaerial temperature exposure is likely far more stressful for mussels at the southern end of their distribution (Angelini et al., 2016; Jost & Helmuth, 2007). However, our acclimation period of 2 weeks is likely sufficient to have helped to separate spatial genetic differences in expression from acclimatory factors (Thompson et al., 2012). Clearly, we also cannot exclude maternal or other transgenerational effects in this experiment. The overall observations, however, reinforce that there are genomic and physiological distinctions between northern and southern populations of *G*. *demissa*, and the transcriptomic responses highlight a small number of candidate gene regions that may be an important part of this variation.

Spatial variation of population responses is our primary guide to understanding how organisms will respond to climate extremes (Des Roches et al., 2018; Hice et al., 2012; Kelly et al., 2012; Palumbi et al., 2019). Similar geographic contrasts in the confamilial mussel *Mytilus californianus* support temperature‐based adaptive responses (Logan et al., 2012). Other *Mytilus* species, *M*. *edulis* and *M*. *trossulus*, experience physiological differences across different geographical regions due to differences in various environmental covariates like temperature and salinity (Telesca et al., 2018). An overall spatial consideration of variation in genomic diversity and plastic response to environment will be key for such an ecologically important organism (Broitman et al., 2021). Given the concordance of intraspecific divergence in *G*. *demissa* (Díaz‐Ferguson et al., 2009) with regional shifts in the genomic composition of many coastal invertebrates (Altman et al., 2013; Bell et al., 2014; Wares, 2002), this mussel may be more generally important as an indicator of how marine environments and patterns of spatial isolation and adaptation tend to interact on this coast. The differences we find here between Georgia and Massachusetts populations of *G*. *demissa* indicate that these populations might respond differently to warming temperatures, and more such integrative rangewide analyses on the Atlantic coast of North America are likely to generate useful awareness of the environmental features that generate and maintain distinct biodiversity.

## AUTHOR CONTRIBUTIONS

**Theresa Erlenbach:** Formal analysis (equal); investigation (equal); methodology (equal); validation (equal); visualization (equal); writing – original draft (equal); writing – review and editing (equal). **John Wares:** Conceptualization (lead); formal analysis (equal); funding acquisition (lead); investigation (equal); methodology (equal); project administration (lead); resources (lead); supervision (lead); validation (equal); visualization (equal); writing – original draft (equal); writing – review and editing (equal).

## FUNDING INFORMATION

This work was supported by a grant from the UGA Office of the Vice President for Research, the Elaine Lutz Fund for Aquatic Biodiversity, and the Odum School of Ecology to John P. Wares, along with an NIH T32 training grant award to TRE (T32GM007103).

## CONFLICT OF INTEREST STATEMENT

There are no competing interests to disclose.

## Supporting information


**Data S1:** Supporting information

## Data Availability

Raw RNA sequence reads can be found under the NCBI BioProject PRJNA729970. Trimmed alignments of gene regions listed in Table 3 are archived at https://doi.org/10.6084/m9.figshare.20813707. All scripts for the transcriptome assembly, differential expression analysis, and population divergence analysis are located online at https://github.com/trm76056/Geukensia_Project.
