# Supplementary material for: Latitudinal variation and plasticity in response to temperature in Geukensia demissa
Source: Ecol Evol. 2023 Feb 24;13(2):e9856. doi: 10.1002/ece3.9856 (PMC9951329; doi:10.1002/ece3.9856)
Supplement: Supplementary file 1 — Data S1: Supporting information [file ECE3-13-e9856-s001.docx]

Appendix 1

**Latitudinal variation and plasticity in response to temperature in *Geukensia demissa***

Theresa Erlenbach^1^ & John P. Wares^1,2^

*^1^Department of Genetics, University of Georgia, Athens, GA, USA*

*^2^Odum School of Ecology, University of Georgia, Athens, GA, USA*

***Figure S1.*** *Temperatures along the coast, relevant to contrasts made in this study. Here each horizontal row of the heatmap represents locations in the overall distributional range of genus Geukensia, including southern congener which is not found within the range of this experiment (Francis & Wares 2022), showing monthly sea surface temperatures (vertical columns) calculated from NOAA ERDDAP data from 2001-2019 across ranges of spatial coastal bins (also see* [*doi.org/10.5670/oceanog.2022.205*](https://doi.org/10.5670/oceanog.2022.205)*). Points “a” and “b” are approximate locations of the sampled populations for this study.*

*
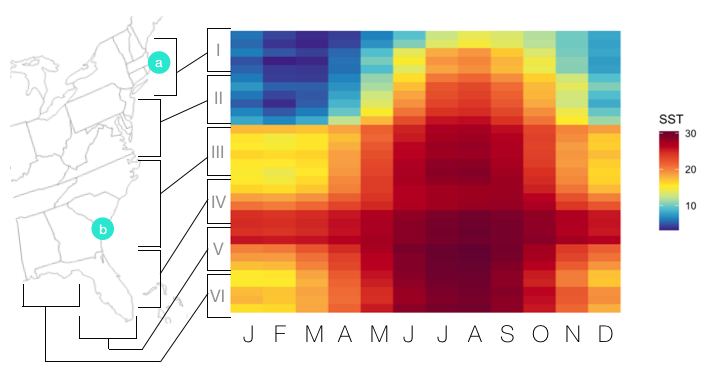
*

***Table S1.*** *Sequenced RNA libraries given their GMNH accession number, population, temperature at harvest, NCBI accession information, RIN, read depth, and mapping efficiency of library to reference transcriptome in bowtie2.*

| **GMNH** | **Population** | **Treatment** | **SRA** | **RIN** | **Reads** | **Mapping Efficiency to GA reference** | **Mapping Efficiency to MA reference** |
| --- | --- | --- | --- | --- | --- | --- | --- |
| 13005 | MA | 25 | SAMN19189390 | 9 | 32767764 | 92.18 | 92.94 |
| 13006 | MA | 25 | SAMN19189391 | 9.6 | 35137562 | 92.04 | 89.85 |
| 13007 | MA | 25 | SAMN19189392 | 8.6 | 41150652 | 92.78 | 91.72 |
| 13009 | MA | 25 | SAMN19189393 | 9.9 | 51310148 | 91.81 | 95.8 |
| 13010 | GA | 25 | SAMN19189378 | 9.5 | 46315292 | 91.27 | 88.87 |
| 13011 | GA | 25 | SAMN19189379 | 9.9 | 36232818 | 90.13 | 90.76 |
| 13012 | GA | 25 | SAMN19189380 | 9.6 | 49080248 | 94.57 | 88.65 |
| 13013 | GA | 25 | SAMN19189381 | 9.3 | 33647952 | 91.13 | 88.71 |
| 13014 | GA | 25 | SAMN19189382 | 9.5 | 30893262 | 92.11 | 90.15 |
| 13015 | MA | 20 | SAMN19189386 | 8.3 | 41888358 | 92.6 | 90.72 |
| 13016 | MA | 20 | SAMN19189387 | 9.4 | 37838804 | 92.67 | 90.71 |
| 13017 | MA | 20 | SAMN1918938 | 9.3 | 39833012 | 92.52 | 89.9 |
| 13019 | MA | 20 | SAMN1918939 | 9.9 | 41294488 | 91.92 | 88.9 |
| 13021 | MA | 30 | SAMN19189383 | 9.2 | 39548532 | 91.32 | 89.42 |
| 13022 | MA | 30 | SAMN19189384 | 9.5 | 40305076 | 93.64 | 91.78 |
| 13023 | MA | 30 | [SAMN19296204](https://dataview.ncbi.nlm.nih.gov/object/SAMN19296204) | 9.2 | 43132678 | 91.56 | 89.96 |
| 13024 | MA | 30 | SAMN19189385 | 9 | 31217380 | 91.48 | 89.74 |
| 13025 | GA | 20 | SAMN19189374 | 9 | 39665934 | 91.49 | 88.52 |
| 13026 | GA | 20 | SAMN19189375 | 9.5 | 35599150 | 90.92 | 88.99 |
| 13027 | GA | 20 | SAMN19189376 | 9.6 | 35565670 | 92.05 | 89.27 |
| 13028 | GA | 20 | SAMN19189377 | 9.8 | 37488016 | 91.3 | 88.55 |
| 13029 | GA | 20 | SAMN19189370 | 9.5 | 33962194 | 91.25 | 89.4 |
| 13030 | GA | 30 | SAMN19189369 | 9.3 | 49446968 | 92.55 | 90.57 |
| 13032 | GA | 30 | SAMN19189371 | 9.4 | 39355864 | 91.04 | 88.98 |
| 13033 | GA | 30 | SAMN19189372 | 9.2 | 41766078 | 91.44 | 89.09 |
| 13034 | GA | 30 | SAMN19189373 | 9.6 | 31758400 | 88.79 | 88.79 |

***Table S2.*** *Calculated VO_2_ in bulk (multiple individual) experiments. Each experiment included 5 individuals held at indicated temperature for 70-90 minutes, with the first time interval removed to allow the system to equilibrate. VO_2_ calculations use either the gross living weight of the individuals or the ethanol-preserved weight of biological tissues from each individual.*

| gross | 20° | 25° | 30° |
| --- | --- | --- | --- |
| MA | 0.0004671 | 0.0005876 | 0.0008798 |
| GA | 0.0004290 | 0.00036855 | 0.0004856 |
| ratio | 1.0887 | 1.5943 | 1.8117 |

| preserved | 20° | 25° | 30° |
| --- | --- | --- | --- |
| MA | 0.0031041 | 0.0039146 | 0.0058471 |
| GA | 0.0032908 | 0.0030717 | 0.0037335 |
| ratio | 0.9432 | 1.2744 | 1.5661 |

**
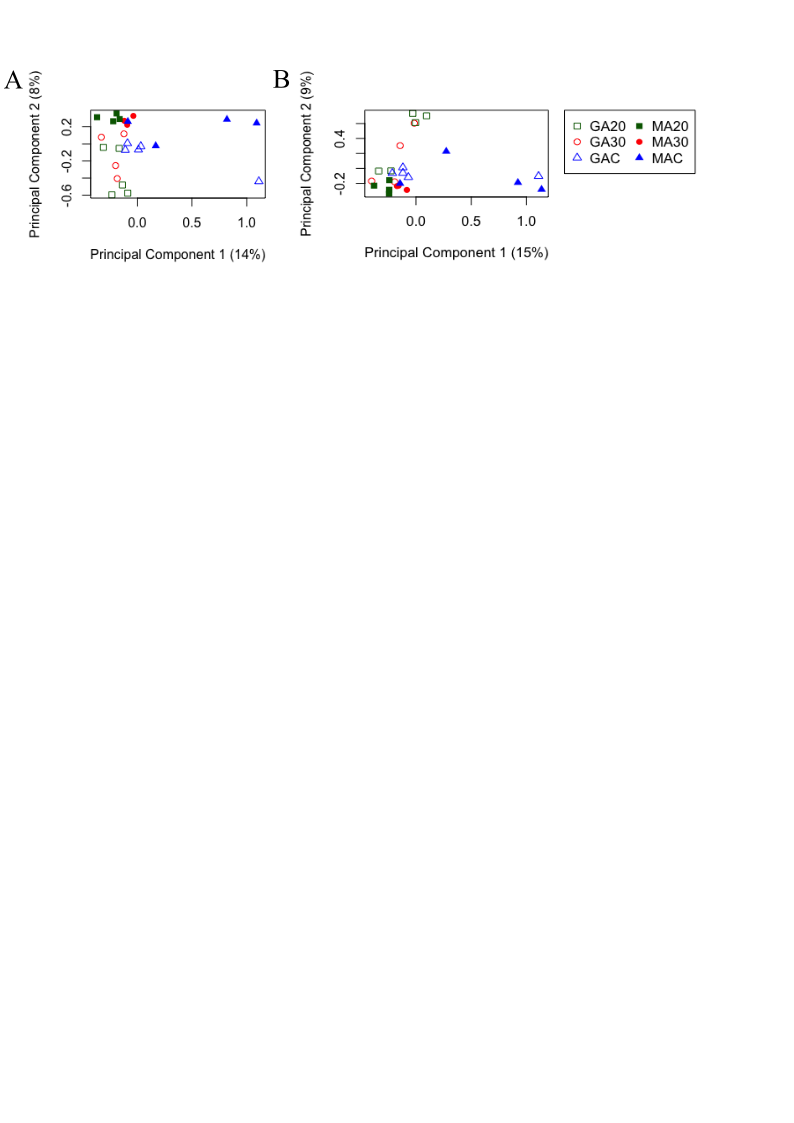
**

***Figure S2****. Principal components plot of RNAseq transcript abundance by library class using either the Georgia control (A) or the Massachusetts control (B) as the reference. Here, GAC and MAC represent the ‘control’ 25° populations that initiated the experiment from GA and MA respectively. Remaining libraries indicate their collection source (GA or MA) and the experimental treatment water temperature (20° or 30°).*


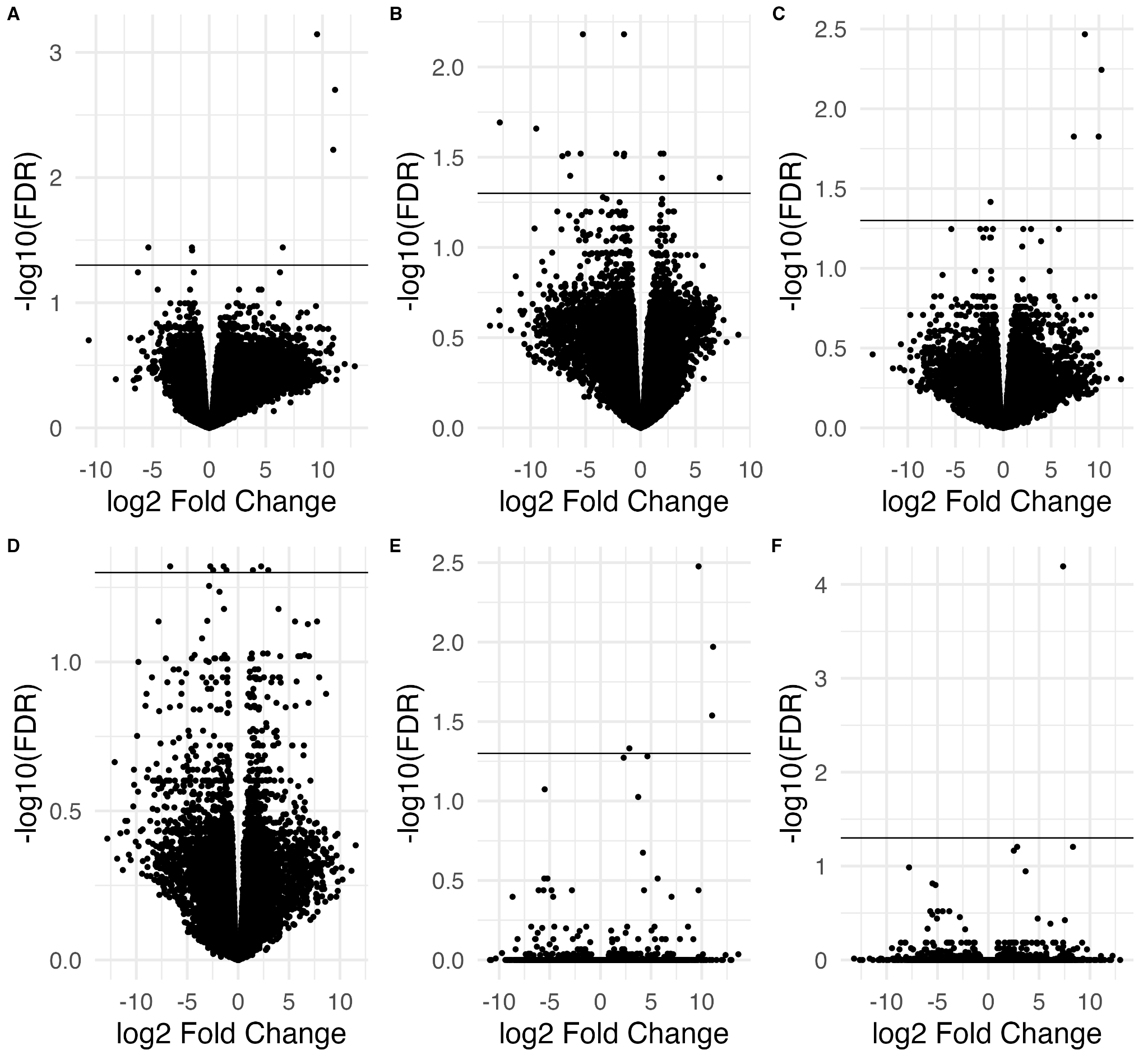


***Figure S3.*** *Differential transcript expression between MA and GA populations. Log2 Fold Change higher than 0 indicates greater expression in the GA population and log2 Fold Change below 0 is higher expression in the MA population. A. Differential expression at the initial common garden temperature of 25° (GAC / MAC) with GAC3 reference transcriptome. B. Differential expression at the initial common garden temperature of 25° (GAC / MAC) with MAC5 reference transcriptome. C. Differential expression at 20° (GA20 / MA20) with GAC3 reference transcriptome. D. Differential expression at 20° (GA20 / MA20) with MAC5 reference transcriptome. E. Differential expression at 30° (GA30 / MA30) with GAC3 reference transcriptome. F. Differential expression at 30° (GA30 / MA30) with MAC5 reference transcriptome. Significance is indicated at FDR < 0.05.*

***Table S3.*** *Orthologs of interest and metabolic loci population genetic statistics, based on MAFFT assemblies and additional curation.*

| **Transcript** | **S_nn_** | **S_nn_ (Additional Curation)** | **d_A_** | **d_A_ (Additional Curation)** | **π per site (GA)** | **π per site (MA)** | **Tajima’s D (GA)** | **Tajima’s D (MA)** | **F_st_** |
| --- | --- | --- | --- | --- | --- | --- | --- | --- | --- |
| OG0016625 | 0.644 | 0.724 | 0.0079 | 0.00099 | 0.0055 | 0.00162 | 0.759 | -0.822 | 0.239 |
| OG0016708 | 0.663 | 0.67667 | 0.0014 | 0.00122 | 0.0095 | 0.00562 | -0.233 | -0.549 | 0.175 |
| OG0016789 | 0.727 | 0.85477 | 0.00097 | 0.00089 | 0.0023 | 0.00607 | -2.098 | -1.08 | 0.197 |
| OG0016809 | 0.649 | 0.5535 | 0.00057 | 0.00044 | 0.0049 | 0.00618 | 0.14 | 0.691 | 0.103 |
| OG0016831 | 0.89 | 0.85667 | 0.0012 | 0.00094 | 0.0051 | 0.00159 | -0.029 | -0.076 | 0.272 |
| OG0016844 | 0.673 | 0.7029 | 0.0023 | 0.00149 | 0.0046 | 0.0065 | -0.466 | -0.178 | 0.321 |
| OG0017382 | 0.773 | 0.892 | 0.0012 | 0.00184 | 0.0033 | 0.0132 | 0.0641 | -2.052 | 0.149 |
| OG0017577 | 0.631 | 0.67333 | 0.00092 | 0.00103 | 0.0109 | 0.00374 | 0.228 | -0.98 | 0.123 |
| OG0017590 | 0.711 | 0.73111 | 0.00054 | 0.00057 | 0.0051 | 0.0036 | -1.308 | 0.203 | 0.154 |
| OG0017668 | 0.677 | 0.68533 | 0.003 | 0.00313 | 0.00086 | 0.0154 | -1.442 | 1.743 | 0.32 |
| Acid Phosphatase | 0.455 | 0.564 | 1.80E-05 | 0.0045 | 0.00464 | 0.0058 | -0.485 | -0.178 | 0.014 |
| ADH | 0.397 | N/A | 1.31E-04 | N/A | 0.0769* | 0.0414 | 2.027 | -1.04 | 0.0537 |
| Albumin | 0.465 | 0.47608 | 0 | 0 | 0 | 0.000409 | NaN | -1.111 | 0 |
| Alkaline Phosphatase | 0.479 | 0.44048 | 0 | 0.00001 | 0.00907 | 0.0118 | -1.105 | -0.742 | 0 |
| Amylase | 0.657* | 0.947 | 8.189e-04* | 0.01522 | 0.00617 | 0.0131 | -1.451 | 0.0188 | 0.651 |
| Aspartate Aminotransferase | 0.625* | N/A | 3.12E-06 | N/A | 0.00145 | 0.00197 | -1.201 | -0.241 | 0.00234 |
| Catalase | 0.455 | N/A | 0 | N/A | 0.0634* | 0 | -2.057 | NaN | 0 |
| Creatine Kinase | 0.521 | N/A | 0 | N/A | 0.00534 | 0.00722 | -1.064 | -0.641 | 0 |
| d-Lactate Dehydrogenase | 0.41 | 0.5 | 0 | -0.0009 | 0.015 | 0.00952 | -0.2 | 0.162 | 0 |
| Glutamate Dehydrogenase | 0.59 | 0.5401 | 1.417e-03* | 0.00113 | 0.0137 | 0.0091 | 2.0117 | 0.0925 | 0.184 |
| Hexokinase | 0.652* | 0.4881 | 4.93E-06 | 0.00015 | 0.0105 | 0.00625 | -0.847 | -1.764 | 0.000634 |
| Iditol Dehydrogenase | 0.562* | 0.52543 | 0 | -0.00006 | 0.00268 | 0.00451 | -0.315 | -0.783 | 0 |
| Isocitrate Dehydrogenase | 0.472 | 0.4 | 7.80E-05 | 0.00016 | 0.00451 | 0.00427* | 0.572 | -0.487 | 0.0238 |
| M7 Lysin | 0.656* | 0.78947 | 2.937e-03* | 0.00347 | 0.00342 | 0.0115 | -1.876 | 0.757 | 0.352 |
| Malate Dehydrogenase | 0.588 |  | 0 |  | 0.0034 | 0.0034 | -1.085 | -0.0855 | 0 |
| Phosphoglucomutase | 0.515 | 0.69667 | 3.61E-05 | 0.00093 | 0.0053* | 0.00355 | 0.181 | 0.291 | 0.0185 |
| Purine Nucleoside Phosphorylase | 0.465 | 0.46462 | 1.18E-06 | 0.00001 | 0.00468 | 0.00346 | -2.055 | -0.67 | 0.000311 |
| 6-Phosphogluconate Dehydrogenase | 0.5236 |  | 4.840e-04* |  | 0.0083 | 0.0109 | -0.614 | 0.423 | 0.198 |
| Glucose-6-Phosphate Dehydrogenase | 0.607 | 0.62667 | 0 | -0.001 | 0.0086 | 0.00927 | -1.262 | 0.615 | 0 |
| Glucose-6-Phosphate Isomerase | 0.375 | 0.275 | 0 | -0.00052 | 0.0103 | 0.012 | -0.701 | -0.529 | 0 |
| Mannose-6-Phosphate Isomerase | 0.461 | 0.51219 | 1.81E-04 | 0.00021 | 0.00418 | 0.00296 | 0.8413 | -0.584 | 0.0747 |
